# Geriatric nutritional risk index as a prognostic factor in patients with recurrent pancreatic cancer

**DOI:** 10.1371/journal.pone.0271073

**Published:** 2022-07-07

**Authors:** Teruhisa Sakamoto, Masahiro Makinoya, Teppei Sunaguchi, Keisuke Goto, Masaki Morimoto, Yuki Murakami, Kozo Miyatani, Takehiko Hanaki, Yuji Shishido, Kyoichi Kihara, Tomoyuki Matsunaga, Manabu Yamamoto, Naruo Tokuyasu, Yoshiyuki Fujiwara

**Affiliations:** Division of Gastrointestinal and Pediatric Surgery, Department of Surgery, School of Medicine, Tottori University Faculty of Medicine, Yonago, Tottori prefecture, Japan; Osaka Medical Center for Cancer and Cardiovascular Diseases, JAPAN

## Abstract

The aim of this study is to investigate the prognostic significance of geriatric nutritional risk index (GNRI) at the time of recurrence in patients with recurrent pancreatic cancer, and the relationship between GNRI and skeletal muscle mass for survival outcomes after recurrence. This study enrolled 77 patients who developed postoperative recurrence. The skeletal muscle mass index (SMI) was used in this study. The patients were divided into a high-GNRI group (n = 36) and a low-GNRI group (n = 41) for the GNRI, and were divided into a high-SMI group (n = 38) and a low-SMI group (n = 39) for SMI. The 2-year post-recurrence overall survival of patients in the high-GNRI group was significantly longer than that of patients in the low-GNRI group (*P* = 0.001). No significant difference for the 2-year post-recurrence OS curves between the high-SMI group and the low-SMI group was observed (*P* = 0.125). Upon stratifying the patients with high GNRI or low GNRI according to SMI, There was no significant difference in the 2-year post-recurrence OS curves between the patients with both high GNRI and high SMI and the patients with high GNRI and low SMI (*P* = 0.399). Similarly, There was no significant difference in the 2-year post-recurrence OS curves between the patients with low GNRI and high SMI and the patients with both low GNRI and low SMI (*P* = 0.256). Multivariate analysis revealed that the GNRI at the time of recurrence was an independent prognostic risk factor in patients with recurrent pancreatic cancer (*P* = 0.019). The GNRI at the time of recurrence is useful for predicting the prognosis in patients with recurrence pancreatic cancer. Skeletal muscle mass at the time of recurrence is not contributed to predict post-recurrence survival of patients with recurrent pancreatic cancer.

## Introduction

Pancreatic cancer remains one of the most lethal malignancies, with a 5-year overall survival (OS) rate of 9% according to the latest report of cancer statistics [1]. One of the reasons for this is that about 80% of patients who undergo pancreatectomy for pancreatic cancer relapse at local or distant sites even if surgical resection with no residual cancer tissue is achieved [2, 3]. Patients with recurrent pancreatic cancer have a median post-recurrence OS of only 10 months even if chemotherapy is administered [4]. Thus, accurate prediction of prognosis in patients with recurrent pancreatic cancer is crucial in determining the optimal treatment for patients’ satisfactory quality of life. To this end, reliable prognostic factors for recurrent pancreatic cancer are indispensable.

As malnutrition is well known to be closely associated with poor survival in patients with cancer, evaluation of nutritional status using nutritional markers has attracted attention in the context of survival outcomes of cancer patients. One of these nutritional markers, the geriatric nutritional risk index (GNRI), is a simple nutritional assessment tool that consists of serum albumin level and the patient’s height and weight. The GNRI has recently been reported as a useful prognostic factor of malignancies in cohort including of not only older but also non-older, although the GNRI was originally developed to predict the risk of nutrition-related mortality and morbidity in hospitalized older patients [5–7]. In pancreatic cancer, preoperative GNRI was reported as a prognostic risk factor [8]. However, the prognostic significance of GNRI in patients who develop recurrence following surgical resection for pancreatic cancer remains unclear.

Against this background, the aim of this study was to evaluate the value of GNRI for predicting prognosis in patients with recurrent pancreatic cancer. In addition, we aimed to examine the effect on survival outcomes of the relationship between GNRI and skeletal muscle mass, which was reported to be closely associated with patients’ function, tolerability to chemotherapy, and prognosis for malignancies, in patients with recurrent pancreatic cancer.

## Materials and methods

### Patients

We retrospectively reviewed the medical records of 124 patients who had undergone pancreatectomy for pathologically proven pancreatic ductal adenocarcinoma at our institution between January 2005 and December 2018. Seventy-nine of 124 (63.7%) patients developed recurrence after surgery. Recurrence after initial surgery was detected by periodical examination such as radiographic imaging including ultrasonography, computed tomography, magnetic resonance imaging and, if necessary, positron emission tomography, and measurement of serum carbohydrate antigen 19–9 (CA19-9) levels. Of 79 patients with recurrent pancreatic cancer, two were excluded because their data were not available. Finally, 77 patients with recurrent pancreatic cancer were enrolled. All patients in this study were of Japanese ethnicity.

Clinical characteristics at the time of recurrence and the pathological findings obtained from initial pancreatic resection were collected from patients’ medical records. These data included the following: age at the time of recurrence, sex, body mass index (BMI) at the time of recurrence, performance status at the time of recurrence, initial site of recurrence, serum albumin level at time of recurrence, neutrophil-to-lymphocyte ratio (NLR) at the time of recurrence, CA19-9 level at the time of recurrence, time to recurrence after pancreatectomy, chemotherapy after recurrence, skeletal muscle mass index (SMI) at the time of recurrence, primary tumor localization, primary tumor size, lymph node involvement at initial surgery, and histological grading for primary tumor. Pathological findings were classified according to the Tumor-Node-Metastasis classification of the International Union Against Cancer (8th edition). Early recurrences were defined as recurrences within 12 months after the initial surgery; late recurrences were those occurring later than 12 months after the initial surgery.

The Tottori University Hospital Ethics Committee approved this study (No.21A125), and the informed consent was waived by the Tottori University Hospital Ethics Committee.

### Geriatric nutritional risk index

Calculation of the GNRI was performed using the following formula: GNRI = [1.489 × serum albumin level (g/L)] + [41.7 × body weight/ideal body weight (kg)]. According to the previous report, the ratio of a patient’s body weight to the ideal body weight was set to 1 when the patient’s body weight exceeded the ideal body weight and the ideal body weight was calculated using the Lorentz equation [7]. The values of serum albumin level and body weight were collected from the data obtained at the time of recurrence.

### Skeletal muscle mass index

The cross-sectional areas of total skeletal muscle mass at the level of the third lumbar vertebra on computed tomography images at the time of recurrence were automatically measured using SYNAPSE VINCENT (Fujifilm, Tokyo, Japan). The skeletal muscle mass index (SMI) was calculated by normalizing the cross-sectional areas of skeletal muscle mass at the level of the third lumbar vertebra to the patients’ height (cm^2^/m^2^) [9].

### Statistical analysis

Differences between two groups were analyzed using chi-squared or Fisher’s exact probability tests for categorical variables, and the Mann-Whitney *U* test for continuous variables with nonparametric distribution. The 2-year post-recurrence survival curves were delineated by the Kaplan–Meier method. The difference between survival curves was estimated by a log-rank test. Receiver operating characteristic (ROC) analysis was used to determine the areas under the curves and the cutoff values of the GNRI, the SMI in this study. Univariate and multivariate analyses were performed using Cox proportional hazards models to determine factors with prognostic significance for 2-year post-recurrence survival. *P* values of less than 0.05 were considered statistically significant. All statistical analyses were performed using IBM SPSS Statistics for Windows (version 24; IBM, Armonk, NY, USA).

## Results

**Fig 1:**
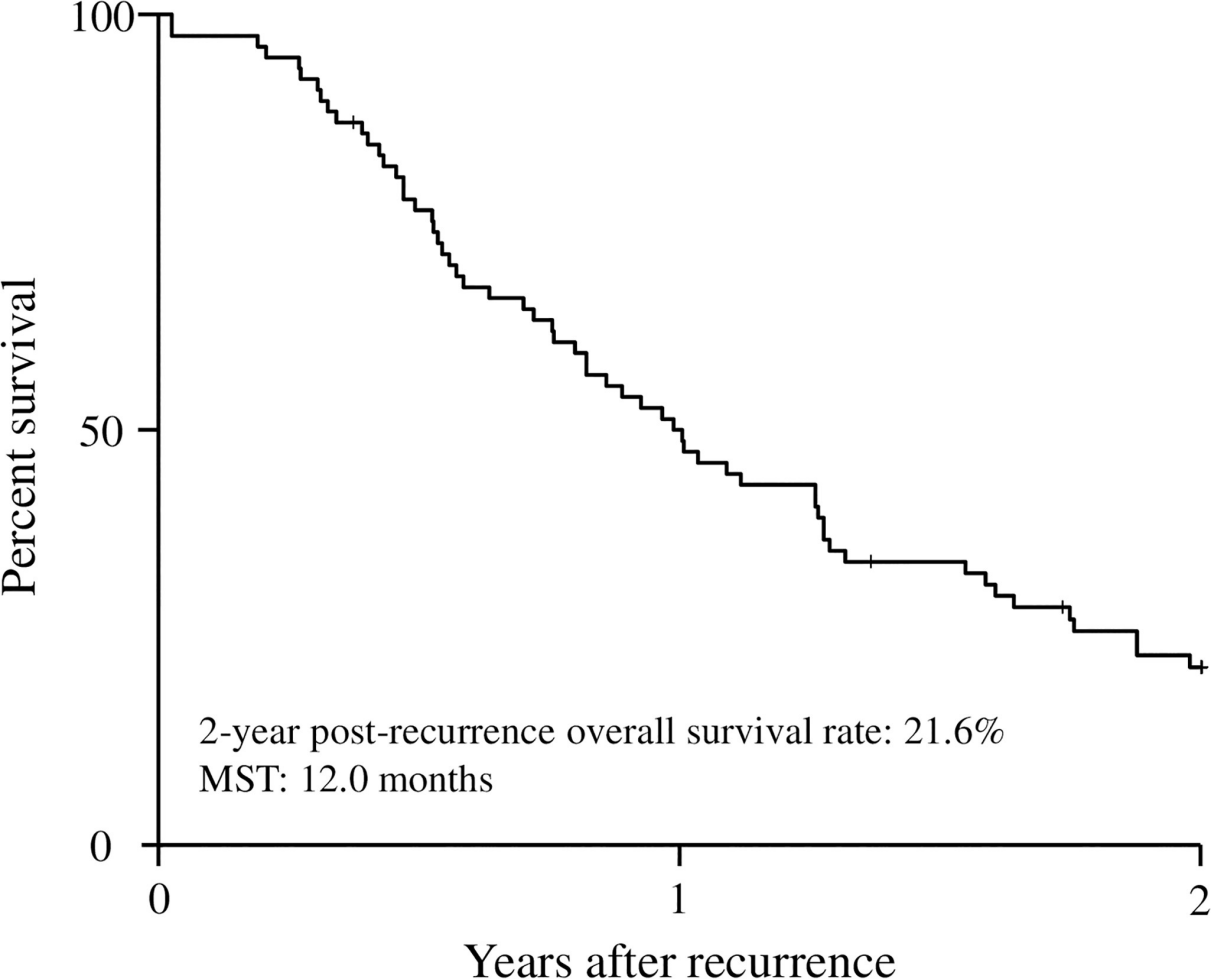
Two-year post-recurrence overall survival curve in the patients with recurrent pancreatic cancer.

**Fig 2:**
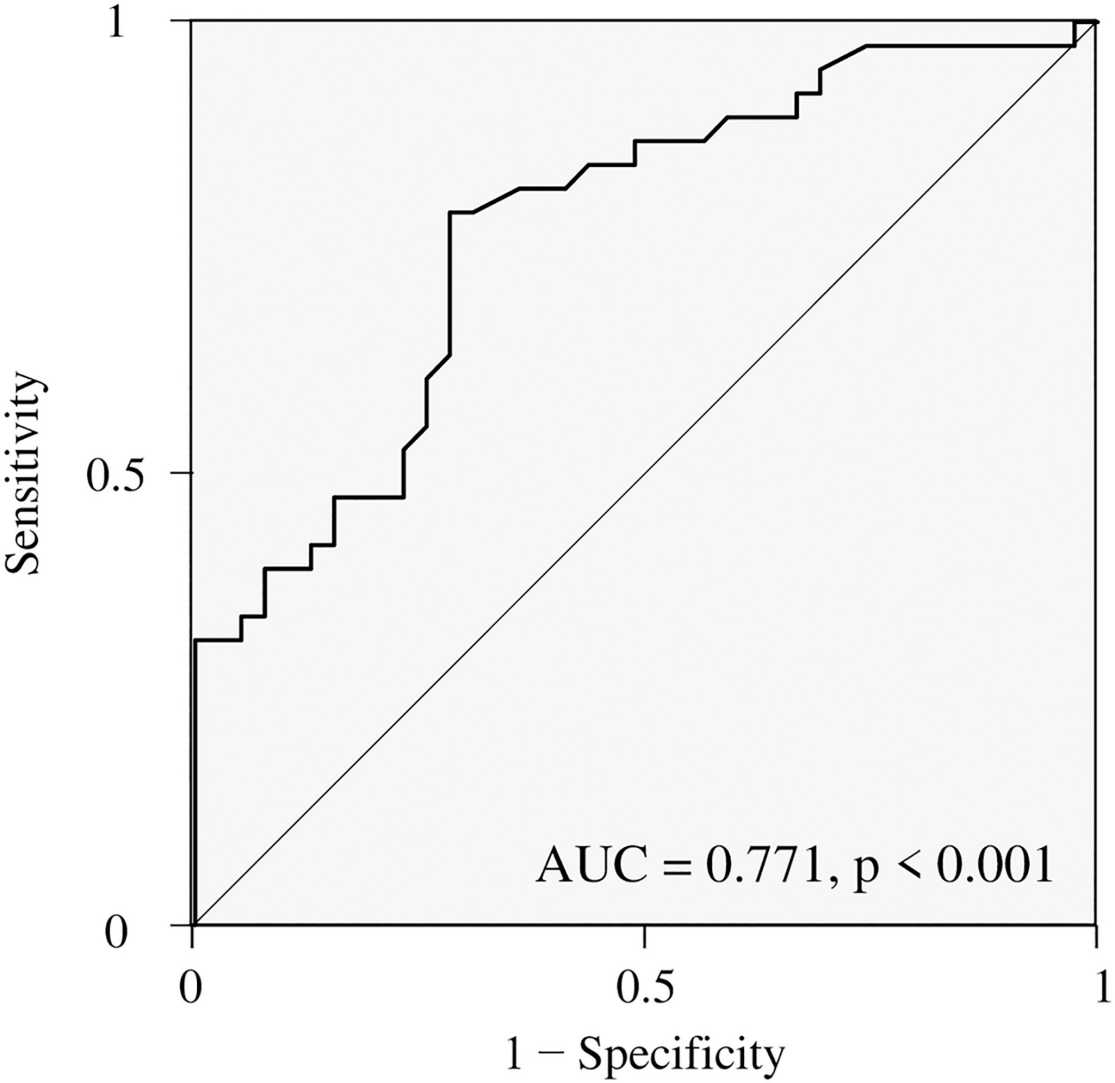
Receiver operating characteristic analysis for predicting 12 months post-recurrence survival.

The median follow-up interval after recurrence was 11.6 months (range, 0.3–84.5 months). The 2-year post-recurrence overall survival rate and median survival time of the whole patients with recurrent pancreatic cancer were 21.6%, 12.0 months, respectively (Fig 1). The mean GNRI of all patients at the time of recurrence was 93.6 ± 11.2 and the mean SMIs were 40.2 ± 7.6 for male patients and 33.8 ± 5.8 for female patients. ROC analysis determined that the optimal cutoff value and the area under the curve of GNRI for predicting 12 months post-recurrence survival were 96.5 and 0.771, respectively (Fig 2). The cutoff values of SMI were determined as 44.4 for men and 30.2for women using ROC analysis for predicting 12 months post-recurrence survival. According to the GNRI and SMI cutoff values, the patients were divided into a high-GNRI group (GNRI ≥ 96.5, n = 36) and a low-GNRI group (GNRI < 96.5, n = 41) for the GNRI, and were divided into a high-SMI group (n = 38) and a low-SMI group (n = 39) for SMI.

Correlations between clinicopathological characteristics and GNRI are summarized in Table 1. Significant correlations were observed between GNRI and sex, BMI at the time of recurrence, performance status at the time of recurrence, serum albumin level at the time of recurrence, NLR at the time of recurrence, time to recurrence after pancreatectomy, chemotherapy after recurrence and SMI at the time of recurrence. BMI and serum albumin level at the time of recurrence in the high-GNRI group were greater than that in the low-GNRI group, and chemotherapy after recurrence was administered to more patients in the high-GNRI group than in the low-GNRI group. By contrast, the incidence of poor performance status, NLR at the time of recurrence and the incidence of early recurrence after pancreatectomy in the low-GNRI group were greater than those in the high-GNRI group. With respect to SMI at the time of recurrence, SMI at the time of recurrence in the low-GNRI group was significantly lower than that in the high-GNRI group. Table 2 shows correlations between clinicopathological characteristics and SMI. Significant correlations were observed between SMI and sex, BMI at the time of recurrence, serum albumin level at the time of recurrence and chemotherapy after pancreatectomy. BMI at the time of recurrence, serum albumin level at the time of recurrence and the incidence of chemotherapy after pancreatectomy in the high-SMI group were greater than those in the low-SMI group. However, there were not significant correlations between SMI and performance status at the time of recurrence, NLR at the time of recurrence, and the incidence of early recurrence after pancreatectomy although significant correlations were observed between GNRI and these characteristics.

**Table 1:** Comparison of clinicopathological characteristics of high-GNRI group and low-GNRI group in patients with recurrent pancreatic cancer.

Characteristics	High-GNRI (≥ 96.5, n = 36)	Low-GNRI (< 96.5, n = 41)	*P* value
Age at time of recurrence (years), median (range)	71 (53–84)	74 (44–85)	0.240
Sex (male), n (%)	19 (52.8%)	31 (75.6%)	0.036
Body mass index at time of recurrence (kg/m^2^), median (range)	21.6 (116.4–27.9)	17.7 (13.3–26.8)	< 0.001
Performance status at the time of recurrence (1 or 2), n (%)	5 (13.9%)	20 (48.8%)	0.001
Primary tumor location (pancreatic head), n (%)	23 (63.9%)	25 (61.0%)	0.792
Primary tumor size (mm), median (range)	28.0 (10.0–85.0)	27.1 (5.0–60.0)	0.745
Lymph node involvement (present), n (%)	20 (55.6%)	31 (75.6%)	0.063
Histological grading (G1^a^), n (%)	19 (52.8%)	18 (43.9%)	0.437
Initial site of recurrence (distant), n (%)	24 (66.7%)	26 (63.4%)	0.765
Serum albumin level at time of recurrence (g/dL), median (range)	4.25 (3.8–5.0)	3.5 (1.5–4.6)	< 0.001
Neutrophil-lymphocyte ratio at time of recurrence, median (range)	1.42 (0.26–5.06)	2.24 (0.41–18.6)	0.002
Serum CA19-9 level at time of recurrence (U/mL), median (range)	214.8 (0.7–1843.0)	123.2 (0.1–20460.0)	0.622
Time to recurrence after pancreatectomy (< 12 months), n (%)	15 (41.7%)	30 (73.2%)	0.005
Chemotherapy after recurrence (present), n (%)	34 (94.4%)	29 (70.7%)	0.008
Skeletal muscle mass index at time of recurrence (low), n (%)	10 (27.8%)	29 (70.7%)	< 0.001

Continuous variables are expressed as the median and range.

^a^G1: well-differentiated.

*GNRI* geriatric nutritional risk index, *CA19-9* carbohydrate antigen 19–9.

**Table 2:** Comparison of clinicopathological characteristics of high-SMI group and low-SMI group in patients with recurrent pancreatic cancer.

Characteristics	High-SMI (n = 38)	Low-SMI (n = 39)	*P* value
Age at time of recurrence (years), median (range)	71 (53–84)	74 (44–85)	0.810
Sex (male), n (%)	18 (47.4%)	32 (82.1%)	0.001
Body mass index at time of recurrence (kg/m^2^), median (range)	21.5 (14.3–27.9)	18.2 (13.3–24.7)	0.001
Performance status at the time of recurrence (1 or 2), n (%)	12 (31.6%)	13 (33.3%)	0.869
Primary tumor location (pancreatic head), n (%)	27 (71.1%)	21 (53.8%)	0.119
Primary tumor size (mm), median (range)	27.5 (15.0–85.0)	28.0 (5.0–60.0)	0.406
Lymph node involvement (present), n (%)	23 (60.5%)	28 (71.8%)	0.296
Histological grading (G1^a^), n (%)	21 (55.3%)	16 (41.0%)	0.211
Initial site of recurrence (distant), n (%)	27 (71.1%)	23 (59.0%)	0.267
Serum albumin level at time of recurrence (g/dL), median (range)	4.1 (2.9–4.7)	3.8 (1.5–5.0)	0.042
Neutrophil-lymphocyte ratio at time of recurrence, median (range)	1.94 (0.26–8.2)	1.70 (0.36–18.6)	0.930
Serum CA19-9 level at time of recurrence (U/mL), median (range)	206.5 (0.7–3446.0)	209.0 (0.1–20460.0)	0.961
Time to recurrence after pancreatectomy (< 12 months), n (%)	18 (47.4%)	27 (69.2%)	0.052
Chemotherapy after recurrence (present), n (%)	36 (94.7%)	27 (69.2%)	0.006

Continuous variables are expressed as the median and range.

^a^G1: well-differentiated.

*SMI* skeletal muscle mass index, *CA19-9* carbohydrate antigen 19–9.

**Fig 3:**
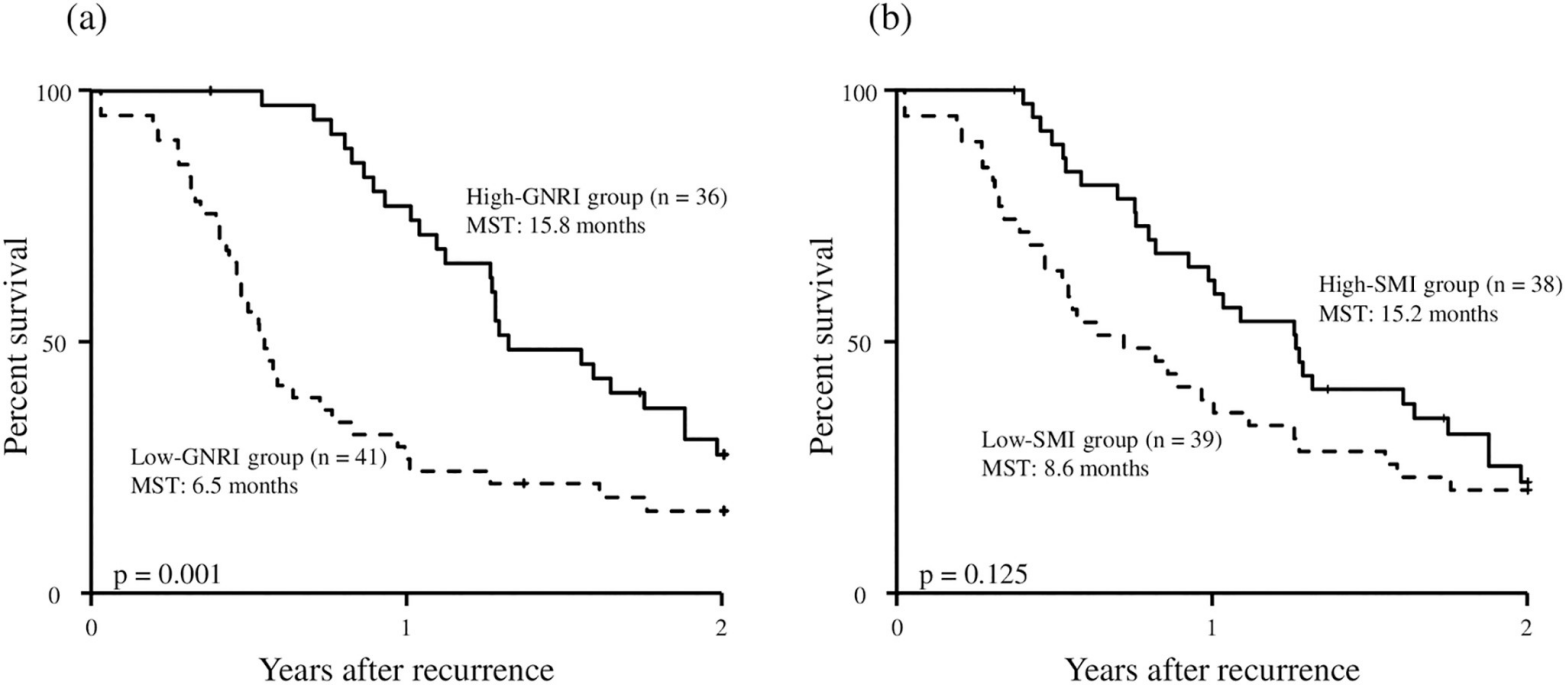
Two-year post-recurrence overall survival curves according to the GNRI (a) and SMI (b) in patients with recurrent pancreatic cancer. *GNRI*, geriatric nutritional risk index; *SMI*, skeletal muscle mass index.

**Fig 4:**
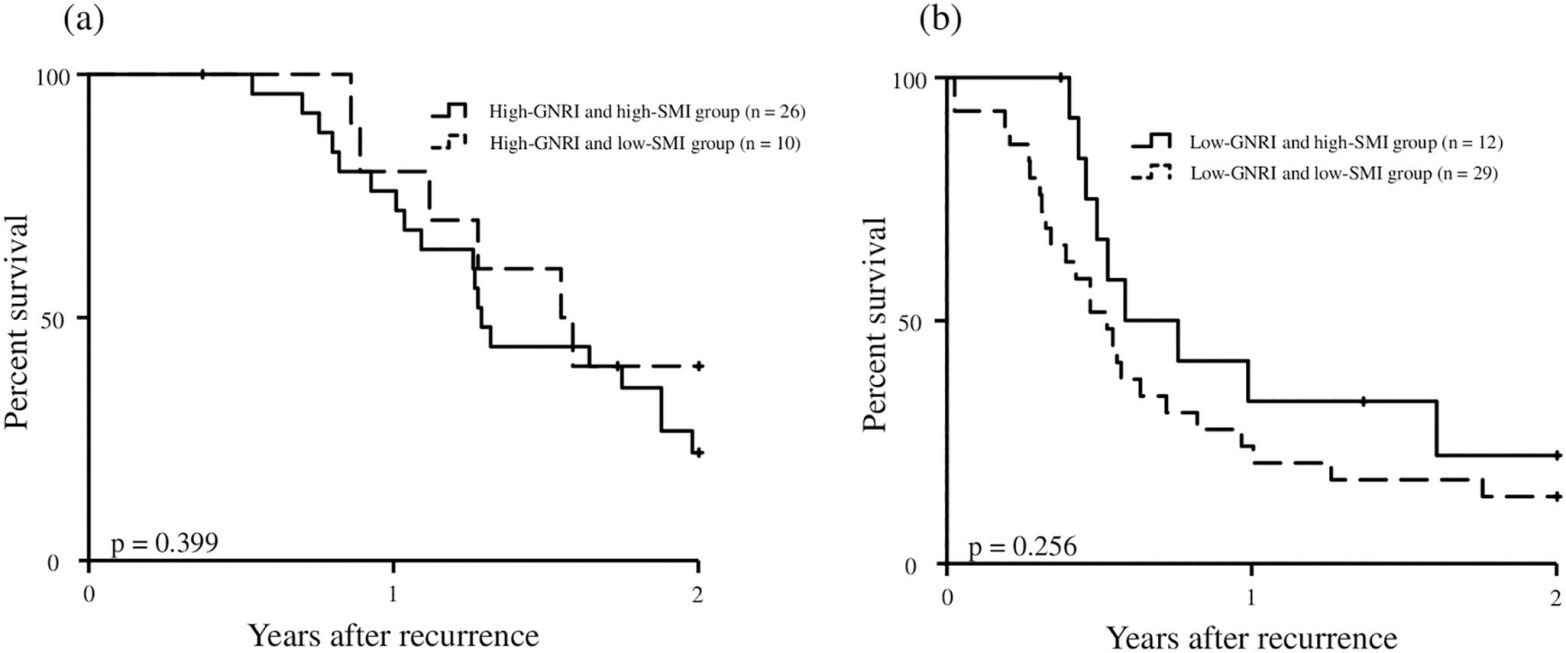
Two-year post-recurrence overall survival curves in the patients with high-GNRI stratified according to SMI (a) and the patients with low-GNRI stratified according to SMI (b). *GNRI*, geriatric nutritional risk index; *SMI*, skeletal muscle mass index.

Fig 3 shows the 2-year post-recurrence OS curves of GNRI or SMI. The 2-year post-recurrence OS rate and median survival time after recurrence were 27.7% and 15.8 months, respectively in the high-GNRI group and 16.5% and 6.5 months, respectively in the low-GNRI group. The 2-year post-recurrence OS curve of the high-GNRI group was significantly superior to that of the low-GNRI group (*P* = 0.001, Fig 3a). The MST in the high-SMI group tended to be longer than that in the low-SMI group, while no significant difference for the 2-year post-recurrence OS curve between the high-SMI group and low-SMI group was observed (*P* = 0.125, Fig 3b). Upon stratifying the patients with high GNRI or low GNRI according to SMI, the patients with high GNRI were stratified into two groups as follows: patients with both high GNRI and high SMI (n = 26), patients with high GNRI and low SMI (n = 10). The 2-year post-recurrence OS curves of these two groups were described in Fig 4a. There was no significant difference in the 2-year post-recurrence OS curves between the two groups (*P* = 0.399). Similarly, the patients with low GNRI were stratified into patients with low GNRI and high SMI (n = 12) and patients with both low GNRI and low SMI (n = 29). The 2-year post-recurrence OS curves of these two groups were described in Fig 4b. There was no significant difference in the 2-year post-recurrence OS curves between the two groups (*P* = 0.256).

Table 3 summarized systemic chemotherapy after recurrence in the patients between high GNRI group and low GNRI group. Sixty-three (81.8%) of 77 patients received systemic chemotherapy after recurrence. No significant correlations between high GNRI group and low GNRI group were observed for the rates of combination chemotherapy or single agent therapy as first-line chemotherapy and the rate of second-line chemotherapy.

**Table 3:** Systemic chemotherapy in the patients with recurrent pancreatic cancer who received chemotherapy between high GNRI group and low GNRI group.

Characteristics	High GNRI group	Low GNRI group	*P* value
First-line chemotherapy			0.458
Combination therapy (n, %)	23 (67.6%)	17 (58.6%)	
Singe agent therapy (n, %)	11 (32.4%)	12 (41.4%)	
Second-line chemotherapy			
Present (n, %)	10 (29.4%)	7 (24.1%)	0.638

Multivariate analysis revealed that GNRI at the time of recurrence was an independent prognostic factor (*P* = 0.019), along with serum CA19-9 level at time of recurrence (*P* = 0.003), NLR at time of recurrence (*P* = 0.003), time to recurrence after pancreatectomy (*P* = 0.002), and chemotherapy after recurrence (*P* < 0.001) (Table 4).

**Table 4:** Univariate and multivariate analyses of prognostic factors for overall survival in patients with recurrent pancreatic cancer.

Variables	Univariate analysis			Multivariate analysis		
	HR	95% CI	*P* value	HR	95% CI	*P* value
Age at time of recurrence (≥ 75 vs. < 75)	0.794	0.465–1.354	0.397			
Sex (male vs. female)	1.240	0.721–2.130	0.437			
Primary tumor location (head vs. body and tail)	0.836	0.495–1.414	0.505			
Primary tumor size (≥ 40.0 mm vs. < 40.0 mm)	1.178	0.504–2.750	0.705			
Histological grading for primary tumor (G1 vs. other)	0.826	0.495–1.379	0.466			
Lymph node metastasis at initial surgery (present vs. absent)	1.618	0.928–2.819	0.090			
Surgical margin at initial resection (present vs. absent)	0.886	0.402–1.954	0.764			
Serum CA19-9 level at time of recurrence (≥ 107.95 U/mL vs. < 107.95 U/mL)	2.998	1.718–5.232	<0.001	2.456	1.365–4.418	0.003
NLR at time of recurrence (≥ 2.23 mm vs. < 2.23 mm)	3.445	2.020–5.877	<0.001	2.317	1.328–4.043	0.003
Time to recurrence after pancreatectomy (< 12 months [early] vs. ≥ 12 months [late])	2.777	1.580–4.883	<0.001	2.579	1.401–4.748	0.002
Initial site of recurrence (distant vs. local)	1.196	0.696–2.054	0.516			
Chemotherapy after recurrence (present vs. absent)	0.396	0.208–0.754	0.005	0.242	0.114–0.516	<0.001
SMI at time of recurrence (high vs. low)	1.488	0.862–2.483	0.128			
GNRI at time of recurrence (< 96.5 vs. ≥ 96.5)	2.351	1.394–3.964	0.001	1.949	1.118–3.398	0.019

*HR* hazard ratio, *CI* confidence interval, *G1* well-differentiated, *CA19-9* carbohydrate antigen 19–9, *NLR* neutrophil-to-lymphocyte ratio, *SMI* skeletal muscle mass index, *GNRI* geriatric nutritional risk index.

## Discussion

This study indicated that GNRI at the time of recurrence was an independent prognostic factor for post-recurrence survival in patients with recurrent pancreatic cancer. On the other hand, it was revealed that skeletal muscle mass body has no significant influence for the prognostic outcome in the patients with recurrent pancreatic cancer.

Cancer recurrence is generally considered an incurable disease. The median post-recurrence survival in patients with recurrent pancreatic cancer is markedly curtailed to within 1 year even if these patients receive chemotherapy [3, 4].

Nutritional status in cancer patients not only reflects biological behaviors arising from cancer but also plays an important role as an indicator of tolerability to cancer treatment, including surgery and chemotherapy, so that nutritional status directly affects prognostic outcome in cancer patients [10–12]. Malnutrition, which is well known to be closely associated with adverse prognostic outcomes in cancer patients, is a multifactorial condition that includes lean body mass, muscle wasting, and inflammation by cancer progression, in some cases leading to cachexia [13]. Furthermore, poor response to chemotherapy and increased susceptibility to chemotherapy-induced toxicity are induced by cancer-related malnutrition [14]. These facts made us to hypothesize that correct evaluation of the nutritional status at the time of recurrence is important for accurate prediction of the prognosis or determination of optimal treatments such as chemotherapy or supportive and palliative care to achieve satisfactory quality of life in patients with recurrent pancreatic cancer whose prognosis was very short. Therefore, the present study was conducted.

The GNRI comprising serum albumin level and body weight and height is reported as a useful prognostic factor in several cancers [15–18]. Also, preoperative GNRI was reported as a prognostic risk factor in pancreatic cancer [8]. In this study, we revealed the prognostic significance of GNRI at the time of recurrence in patients with pancreatic cancer, although the usefulness of GNRI in patients with recurrent pancreatic cancer had remained unclear. Serum albumin is one of the most relevant indicators of nutritional status and the acute phase of inflammation. Serum albumin levels decrease in the presence of inflammation, and the production of albumin is inhibited by pro-inflammatory mediators [19–21]. Inflammatory cytokines, such as interleukin-1, interleukin-6, and tumor necrosis factor α, not only promote angiogenesis, tumor growth but also impair cell-mediated immune response [22–24]. Therefore, as the decline of serum albumin reflects tumor progression through cancer-related inflammation, serum albumin levels are closely correlated with prognosis in patients with cancer. Cachexia and its’ development are induced by cancer-related systemic inflammation [25]. NLR is an inflammatory indicator recognized as a prognostic factor in patients with cancer and high NLR offers poorer prognosis to patients with cancer [26, 27]. Inflammatory cytokines, such as IL-6, tumor necrosis factor and vascular endothelial growth factor VEGF produced by neutrophils activation may enhance tumor growth. These cytokines, particular Interleukin-6 have been also shown to promote tumorigenesis by regulating multiple signaling pathways related to apoptosis, proliferation, angiogenesis, invasiveness and metastasis [28, 29]. This study demonstrated that serum albumin at the time of recurrence in low-GNRI group significantly lesser than that in high-GNRI group, and conversely, NLR at the time of recurrence was significantly greater in than that in high-GNRI group. These results indicated that tumor progression was more aggressive in low-GNRI group, which could explain that the patients with low-GNRI had poorer prognosis than with high-GNRI.

Sarcopenia is considered to be debilitating disorders of skeletal muscle and there is a closely relationship between sarcopenia and prognosis in patients with cancer. Loss of Skeletal muscle mass leads to patients with advanced cancer poor prognosis and several reports said that sarcopenia defined by SMI was a useful prognostic factor in advanced cancers [30–32]. Sarcopenia is featured by the absence of inflammation [33]. However, inflammatory cytokines released from cancer tissue were reported to promote skeletal muscle wasting or inhibit myogenesis in patients with advanced cancer [25]. Furthermore, skeletal muscle loss impaired immune responses of cancer patients because counts of immunocytes pooled in skeletal muscle decreased [34]. These could explain the prognostic relationship between skeletal muscle mass volume and patients with advanced cancer. However, our results showed that no significant correlations were observed between SMI and NLR or performance status at the time of recurrence that were closely associated with prognosis in patients with cancers, and that skeletal muscle mass measured at the time of recurrence did not affect post-recurrence survival in the patients with recurrent pancreatic cancer. Although skeletal muscle mass had been reported to affect poor prognosis significantly to patients with advanced cancer, several recent reports said that skeletal muscle mass did not affect survival of advanced cancer patients [35–37]. Takeda et al. reported that metastatic pancreatic cancer patients aged ≥75 with weight loss greater than 5%, or weight loss greater 2% with either a BMI of < 20 or the presence of decline skeletal muscle mass had significantly shorter progression free survival than other older advanced pancreatic cancer patients, and that overall survival of these patients tender to be shorter than others, although this was not significant. In addition, with respect to SMI, they reported that SMI did not affect progression free survival and overall survival of older patients with advanced pancreatic cancer. Furthermore, in their report, body weight loss was also associated with early discontinuation and reduced relative dose intensity of chemotherapy in patients who received first line chemotherapy and modified Glasgow prognostic score, which was a nutritional score consisted of albumin and C-reactive protein used as predictive prognostic factor of patients with cancer, was an independent prognostic factor [37]. These reports support our results that the GNRI at the time of recurrence was significantly associated with post-recurrence survival in patients with recurrent pancreatic cancer, while the SMI had less impact on their survival after recurrence. This might be assumed that decrease of muscle volume is strongly affected by not only cancer-related response but also age-related response or physical burden due to initial pancreatic surgery, which is differ from the GNRI affected by cancer-related inflammation mainly.

This study has several limitations. First, it was a retrospective study with a limited sample size of only Asian individuals from a single institution, which might have limited the generalizability of the findings. Second, although the cutoff value of the GNRI was determined using ROC analysis in this study, the cutoff value of GNRI with respect to the prognosis for cancer patients has not been established. Therefore, the optimal cutoff value of the GNRI for post-recurrence survival in patients with recurrent pancreatic cancer remains unclear. Third, the selection of first- line systemic chemotherapy was not unified. This have led to bias. Therefore, a multicenter, larger-scale study is needed to verify the impact of GNRI as a prognostic factor on recurrent pancreatic cancer.

## Conclusions

The GNRI at the time of recurrence is useful for predicting the prognosis after recurrence in patients with recurrent pancreatic cancer. Skeletal muscle mass at the time of recurrence has less impact on post-recurrence survival of patients with recurrent pancreatic cancer. The GNRI at the time of recurrence might contribute to determine the optimal treatment for patients’ satisfactory quality of life in patients with recurrent pancreatic cancer.
